# Integrative proteomic and gene expression analysis identify potential biomarkers for adjuvant trastuzumab resistance: analysis from the Fin-her phase III randomized trial

**DOI:** 10.18632/oncotarget.5080

**Published:** 2015-09-03

**Authors:** Amir Sonnenblick, Sylvain Brohée, Debora Fumagalli, Françoise Rothé, Delphine Vincent, Michael Ignatiadis, Christine Desmedt, Roberto Salgado, Nicolas Sirtaine, Sherene Loi, Patrick Neven, Sibylle Loibl, Carsten Denkert, Heikki Joensuu, Martine Piccart, Christos Sotiriou

**Affiliations:** ^1^ Breast Cancer Translational Research Laboratory, Institut Jules Bordet, Université Libre de Bruxelles, Brussels, Belgium; ^2^ Pathology Dept, Institut Jules Bordet, Université Libre de Bruxelles, Brussels, Belgium; ^3^ Division of Cancer Medicine and Research, Peter MacCallum Cancer Centre, East Melbourne, Victoria, Australia; ^4^ Multidisciplinary Breast Center, KULeuven, University Hospitals, Belgium; ^5^ German Breast Group, Neu-Isenburg and Sana-Klinikum, Offenbach, Germany; ^6^ Institute of Pathology, Charité Hospital, Campus Mitte, German Cancer Consortium (DKTK), Berlin, Germany; ^7^ Department of Oncology, Helsinki University Central Hospital and Helsinki University, Helsinki, Finland

**Keywords:** trastuzumab resistance, AnnexinA1 (ANXA1), Fin-her, randomized trial

## Abstract

Trastuzumab is a remarkably effective therapy for patients with human epidermal growth factor receptor 2 (HER2) - positive breast cancer (BC). However, not all women with high levels of HER2 benefit from trastuzumab.

By integrating mRNA and protein expression data from Reverse-Phase Protein Array Analysis (RPPA) in HER2-positive BC, we developed gene expression metagenes that reflect pathway activation levels. Next we assessed the ability of these metagenes to predict resistance to adjuvant trastuzumab using gene expression data from two independent datasets.

10 metagenes passed external validation (false discovery rate [fdr] < 0.05) and showed biological relevance with their pathway of origin. These metagenes were further screened for their association with trastuzumab resistance. An association with trastuzumab resistance was observed and validated only for the AnnexinA1 metagene (ANXA1). In the randomised phase III Fin-her study, tumours with low levels of the ANXA1 metagene showed a benefit from trastuzumab (multivariate: hazard ratio [HR] for distant recurrence = 0.16[95%CI 0.05–0.5]; *p* = 0.002; fdr = 0.03), while high expression levels of the ANXA1 metagene were associated with a lack of benefit to trastuzmab (HR = 1.29[95%CI 0.55–3.02]; *p* = 0.56). The association of ANXA1 with trastuzumab resistance was successfully validated in an independent series of subjects who had received trastuzumab with chemotherapy (Log Rank; *p* = 0.01).

In conclusion, in HER2-positive BC, some proteins are associated with distinct gene expression profiles. Our findings identify the ANXA1metagene as a novel biomarker for trastuzumab resistance.

## INTRODUCTION

The human epidermal growth factor receptor 2 (HER2) gene encodes a tyrosine kinase receptor that controls important signal transduction pathways in breast cancer [1]. Amplification and overexpression of the HER2 gene occur in approximately 20% of breast cancers and are associated with an aggressive clinical phenotype [2]. Trastuzumab, a humanized monoclonal antibody that targets HER2, has shown exceptional efficacy in the treatment of breast cancer [3]. In the adjuvant treatment of patients with breast cancer, five randomised trials have shown significant benefit with trastuzumab, reducing the rate of recurrence by approximately 50%, and the rate of death by approximately 30% [4–7].

While trastuzumab is a remarkably effective therapy in patients with HER2-positive breast cancer, not all women with high levels of HER2 respond to trastuzumab. The proposed mechanisms of trastuzumab action are diverse, but it is agreed that trastuzumab must be able to bind the HER2 extracellular domain and, in so doing, it inhibits the PI3K/Akt pathway, which leads to subsequent inhibition of cell proliferation and survival [8]. Mechanisms for primary trastuzumab resistance include compensatory signalling by other cell surface receptors, such as EGFR/HER3, and constitutive activation of downstream effectors or cross-talk pathways [8–10].

The Tumor Cancer Genomic Atlas project (TCGA) performed in-depth analyses of primary breast cancers using five different platforms (DNA copy number arrays, DNA methylation, mRNA arrays, microRNA sequencing and reverse-phase protein arrays) [11]. Its ability to integrate information across different platforms provided key insights; however, the practical translational interpretation of the data has been difficult, as the clinical part of the study was not performed in a randomised controlled manner. Here we developed an in silico bioinformatics approach integrating proteomic and gene expression data to uncover novel biomarkers associated with trastuzumab benefit. By integrating RNA and protein expression data from Reverse-Phase Protein Array Analysis (RPPA) in HER2+ breast cancer from the TCGA repository, we identified several gene expression metagenes that reflect the level of pathway activation according to the expression of proteins and/or phosphorylated-proteins relevant in cancer. Among the identified metagenes, only AnnexinA1 (ANXA1) was successfully associated with trastuzumab benefit in two independent datasets of subjects who had received trastuzumab in the adjuvant setting.

## RESULTS

### Identification of the RPPA-based gene expression metagenes

**Figure 1:**
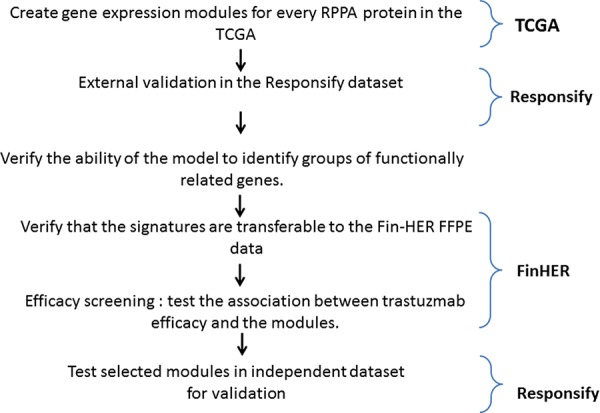
Metagene building and assessment procedure

**Figure 2:**
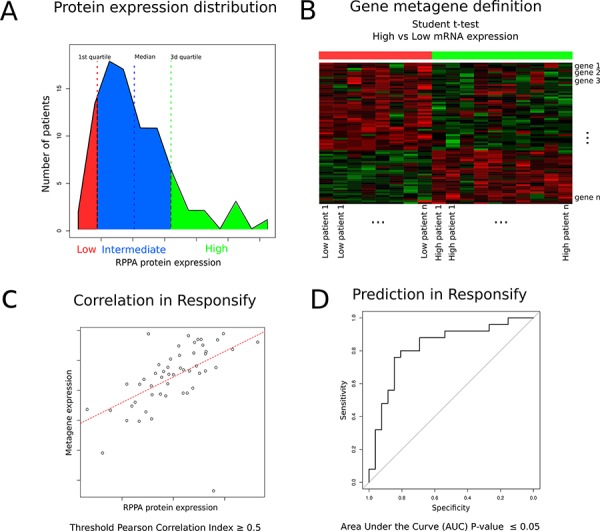
Schematic description of metagene building and assessment **A.** For each (phospho-) protein tested with RPPA in the TCGA repository, generation of groups of patients expressing low (red), intermediate (blue) and high (green) protein levels. **B.** Metagene computation based on significant differential expression of genes discriminating each of the low and high expression groups (*t*-test). **C.** External validation of each metagene by evaluating whether its expression correlates (Pearson Correlation Index > 0.5) with the RPPA expression values in the independent “Responsify” dataset. **D.** ROC curve demonstrating predictive ability of the different metagenes as continuous variables to predict RPPA proteomic data in HER2-positive tumors in the external Resposnify repository. 10 metagenes passed external validation (fdr < 0.05) and were further screened for their association with trastuzumab benefit.

We first retrieved the clinico-pathological and the normalised gene and protein expression data (gene expression microarray and RPPA, respectively) from the public TCGA database of the TCGA (Figure 1 flow chart) [11]. Eighty-seven samples with available gene expression and RPPA data were HER2-positive. For each RPPA (phospho-) protein tested, we classified samples into three groups according to their protein expression levels. The patients expressing the protein at (1) low (lower quartile), (2) intermediate and (3) high (upper quartile) levels were classified into low, intermediate and high RPPA groups respectively (Figure 2A). Those thresholds were defined arbitrarily, as there is no clear cut-off reflecting the protein levels in the cell and we thought those were good indicators of the high or low protein expression level. By computing the genes with significant differential expression (*t*-test fdr ≤ 0.05) between the low and the high RPPA expression groups of each protein, we derived metagenes with expression levels that could potentially mirror protein activation levels (Figure 2B).

We consequently identified 139 gene expression metagenes corresponding to each of the tested RPPA proteins. Each metagene was externally validated using an independent dataset of HER2-positive BC (the Responsify dataset), for which RPPA and gene expression data were available. 10 metagenes passed an external validation step using the independent novel responsify set (Pearson correlation index greater than 0.5 and significant predictive performance with area under the curve of the receiver operating curves-AUC fdr ≤ 0.05) (Figure 2C– 2D, supplementary Tables 1 and 2).

Although we did not develop these metagenes to replace proteomic analysis, these results confirmed that, in HER2-positive BC, some proteins are associated with distinct gene expression profiles.

### Biological relevance of the RPPA-based metagenes

**Figure 3:**
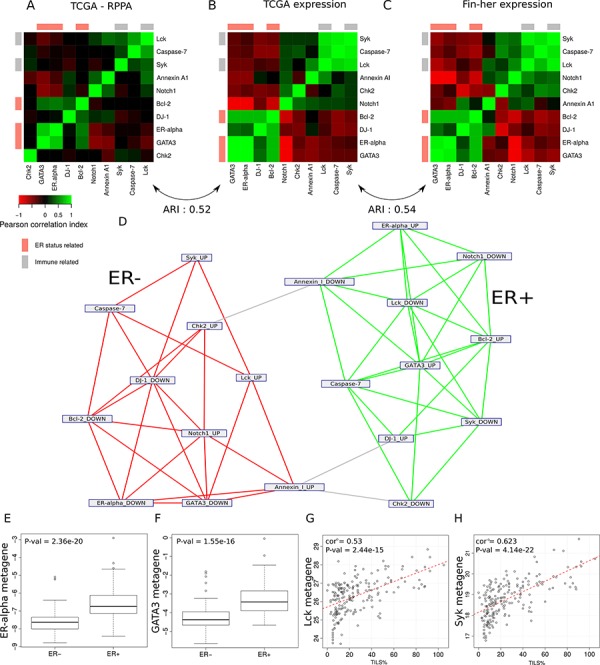
Ability of the model to identify groups of functionally related genes in the different sets **A–C.** Heat map representation of the correlations between (A) the protein expression values within the TCGA dataset, (B) the metagene expression within the TCGA dataset, (C) the metagene expression within the Fin-her dataset. Cells are coloured according to Pearson correlation coefficient values, with green indicating positive correlation and red negative correlation. **D.** Network representation of the metagenes. Each node represents the genes up- or down-regulated in the metagene. Edges show metagenes sharing a significantly high number of genes. The use of a network clustering algorithm showes the tendency of these metagenes to cluster together according to their ER-status. **E–F.** (E) ER-alpha and (F) GATA3 RPPA-based metagenes known to be associated with ER-positive tumours were able to predict pathological ER status in the Fin-her dataset. **G–H.** Correlation of the immune related metagenes (G) Lck and (H) Syk with the percentage of TILs in the Fin-her dataset.

We next sought to assess the biological relevance of the RPPA-based metagenes using different approaches. A comparison of the metagenes to the reference classes of the Gene Ontology and the mSigDB signatures of the Broad Institute [12] showed that the metagenes included a high number of functionally related genes that could capture the corresponding protein/pathway activation (Supplementary Table 3-4). The comparison of the RPPA-based metagene patterns with the RPPA proteomic data demonstrated a significant similarity (Rand Index = 0.52). The RPPA-based gene expression metagenes clustered in related functional groups similar to the ones generated using the RPPA data (Figure 3A–3B). Finally, by applying a clustering algorithm to a network built by linking all significantly intersecting metagenes, we identified two main sub-networks according to their ER status, namely ESR1up/GATA3up and ESR1down/GATA3down, respectively (Figure 3D). This is in line with the TCGA gene expression analyses, which showed that HER2 positive tumors were clustered into two similar major groups based on their ER status [11].

Similar results were found using the Fin-her FFPE-derived dataset (Figure 3C). The comparison of the RPPA-based metagene patterns derived from the TCGA dataset with the ones generated using the Fin-her dataset showed a high similarity (Rand index = 0.54) (Figure 3C). Of note, the ESR1 and GATA3 RPPA-based metagenes known to be associated with ER-positive tumours were significantly correlated with ER status determined by IHC (*p* = 2.36e^−20^, *p* = 1.55e^−16^ respectively) (Figure 3E,3F), while lymphocyte-specific protein tyrosine kinase (Lck) and spleen tyrosine kinase (Syk) RPPA-based immune-derived metagenes significantly correlated with TILs levels (Lck; *R* = 0.53, *p* = 2.44e-^15^ and Syk; *R* = 0.62, *p* = 4.14e^−22^) (Figure 3G,3H). Altogether, these results demonstrate that RPPA-based gene expression metagenes mirror the proteomic status of the samples for selected pathways.

### RRPA-based gene expression association with trastuzmab benefit

**Figure 4:**
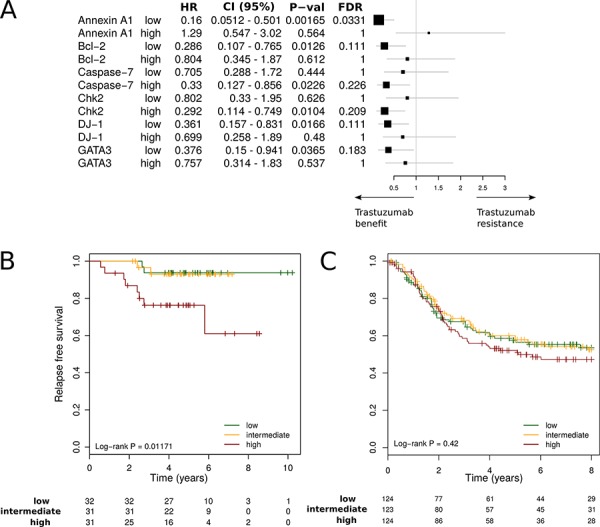
Interaction between RPPA-based metagenes and trastuzumab efficacy **A.** Forest plots in the Fin-her dataset indicate Multivariate Cox regression hazard ratios (HRs) and 95% confidence intervals (CIs) for trastuzumab benefit for distant disease-free survival (DDFS) according to the metagene. **B, C.** Kaplan-Meier plots comparing high versus intermediate and low metagene levels in adjuvant trastuzumab-treated patients in the Responsify dataset (B) and a cohort of HER2 positive patients which did not receive adjuvant trastuzumab (C).

The 10 metagenes (Supplementary Table 2) that passed the external validation were further screened for their association with trastuzumab benefit using gene expression dataset from the prospective randomised Fin-her trial. The patients with available gene expression data who were involved in our sub-study were representative of the entire population, and there were no substantial differences between their patient and tumour characteristics and patients not included (Table 1 and figure S1). An association (arbitrary multivariate Cox regression cut-off *p* ≤ 0.05) with benefit from trastuzumab was observed for six metagenes, but only AnnexinA1 (ANXA1) was found significant after correcting for multiple comparisons (fdr = 0.03) (Figure 4A). Forest plot analysis demonstrated that tumours expressing low levels of the metagene derived from ANXA1 (dichotomised at the median) showed a benefit from trastuzumab (multivariate: hazard ratio [HR] = 0.16 [95% CI 0.05–0.5] *p* = 0.002; fdr = 0.03). Conversely, high expression levels of the ANXA1 metagene were associated with a lack of benefit to trastuzumab (HR = 1.29 [95% CI 0.55–3.02]; *p* = 0.56) (Figure 4A). ANXA1 association with reduced benefit from trastuzumab was confirmed on the independent Responsify dataset of HER2-positive BC patients treated with trastuzumab in the adjuvant setting (Log Rank *p* = 0.01) (Figure 4B). By contrast, in a cohort of HER2 positive patients which did not receive adjuvant trastuzumab (retrieved from gene expression databases previously described in [13]), the ANXA1 metagene had no significant prognostic value (Log Rank; *p* = 0.42), suggesting that ANXA1 is predictive rather than prognostic. We therefore sought to further explore ANXA1 metagene predictive ability in the Fin-Her dataset using Cox univariate and multivariable analysis as continuous variable and interaction tests. Interestingly, the ANXA1 metagene provided independent predictive information for patients with ER-negative breast cancer with a significant multivariate interaction test of *p* = 0.005 (Table 2). As ANXA1 metagene was found to be negativity associated with ER (*R* = −0.3, *p* < 0.001), it suggests that in the HER2+/ER- subgroup, ANXA1 metagene may identify patients with trastuzumab resistance.

**Table 1:** Fin-her patient characteristics

Characteristic	subclass	Whole HER2 positive cohort (*N* = 231)	Cohort with Gene expression (*N* = 202)	*P* value	With trastuzumab (*N* = 100)	No trastuzumab (*N* = 102)	*P* value
Age	< 50	108	94	1	51	43	0.26
	>= 50	123	108		49	59	
Tumor stage	T1	81	74	0.94	33	41	0.49
	T2	135	115		60	55	
	T3	14	12		7	5	
	NA	1	1		0	1	
Nodal status	Negative	37	29	0.73	80	93	0.04
	1–3	194	173		20	9	
Histological grade	1	5	5	1	3	2	0.57
	2–3	220	191		93	98	
	NA	6	6		4	2	
ER status	Positive	109	97	0.93	46	51	0.66
	Negative	122	105		54	51	
Histology	Ductal	208	181	1	87	94	0.56
	Lobular	21	19		11	8	
	NA	2	2		2	0	

**Table 2:** Cox univariate and multivariable analysis of ANXA1 metagene treated as a continuous variable, in the Fin-her study

	DDFS prognostic value of ANXA1 metagene (No trastuzumab)						DDFS prognostic value of ANXA1 metagene (trastuzumab)						
	Univariate			Multivariate			Univariate			Multivariate			*P* interaction
	HR	CI 95%	*P*	HR	CI 95%	*P*	HR	CI 95%	*P*	HR	CI 95%	*P*	
All	0.76	[0.52 – 1.1]	0.14	0.78	[0.53 – 1.15]	0.53	1.06	[0.641 – 1.75]	0.81	1.15	0.68 – 1.91	0.59	0.3
ER+	0.74	[0.41 – 1.33]	0.31	0.7	[0.36 – 1.35]	0.36	0.52	[0.28 – 0.96]	0.03	0.66	0.32 – 1.35	0.26	0.41
ER-	0.74	[0.46 – 1.2]	0.22	0.78	0.44 – 1.38]	0.44	2.4	[1.36 – 4.45]	0.0028	2.0	0.97 – 4.15	0.06	0.005

For multivariate analysis, we considered the following variables: age, tumor size, grade, nodal status, and ER status. The interaction test is for the multivariate analysis

## DISCUSSION

In the present study, we integrated RPPA and gene expression data to systematically interrogate protein pathway activation. To our knowledge, this is one of the first studies to use such a methodology and to demonstrate that RPPA-based gene expression metagenes may be able to reflect proteomic activation status using FFPE samples.

The same general approach was used in two studies to link upstream signalling pathways to downstream transcriptional response by exploiting RPPA and mRNA expression in breast cancer. While demonstrating the robustness of the approach, both studies lacked external confirmatory prospective randomised cohorts and datasets [14, 15].

One of the strengths of our study is the use of gene expression data derived form a prospective clinical trial that randomised patients with HER2-positive breast cancer to receive treatment with or without trastuzumab. Moreover, the Responsify dataset, in which both RPPA and RNA expression data were available for patients with HER2-positive breast cancer who were treated with trastuzumab in the adjuvant setting with the more standard duration of one year, enabled us to perform an external validation of the data in this specific subset of patients.

For the first time, we have demonstrated an association between ANXA1 and trastuzumab resistance. ANXA1 is a 37 kDa calcium and phospholipid binding protein that is involved in inflammatory processes, cell proliferation, and the regulation of cell death and carcinogenesis [16]. Previous research using tissue microarray analysis has shown that in some breast cancers, ANXA1 loss leads to faster tumour growth [17] and cancer progression [18]. ANXA1 expression has also been found to be associated with a highly invasive basal-like breast cancer subtype and to promote metastasis formation by enhancing the TGFβ/Smad signalling pathway, which in turn facilitates an epithelial mesenchimal transition switch [19]. Our results regarding DJ-1 metagene, a negative regulator of PTEN that indirectly activates pAkt [20], are also in line with previous pre-clinical data supporting the role of the Akt/mTOR pathway with trastuzumab resistance [21, 22]. However, this association was not validated in the independent Responsify dataset and did not pass the false discovery rate threshold.

Overall, our finding that RPPA-based, gene expression metagenes predict lack of benefit to trastuzumab through ANXA1 raises new questions regarding the postoperative management of HER2-positive breast cancer. If confirmed by future prospective, randomised, controlled studies (such as ALTTO or APHINITY) this RPPA-based gene expression signature could be used to direct the rationale for adjuvant treatment and research in HER2-positive breast cancer.

## MATERIALS AND METHODS

### Study cohorts

Our study used two study cohorts, one from the Fin-her trial, the other from the Responsify study. The Fin-her trial is a multicentre phase 3 randomised adjuvant breast cancer trial that enrolled 1010 patients [4, 23] (The trial identifier is ISRCTN76560285, supplementary figure 1 [CONSORT diagram]). The women were randomly assigned to receive three cycles of docetaxel or vinorelbine, followed by three cycles of fluorouracil, epirubicin, and cyclophosphamide. Out of the 1010 patients, 232 patients with HER2-positive breast cancers were further randomised to 9 weeks of trastuzumab or no trastuzumab. Two-hundered and two of the 232 HER2-positive tumour samples collected had sufficient RNA with good quality for gene expression analysis. The clinical pathological characteristics of the HER2-positive patients with available gene expression data (*n* = 202) were compared with the general series (*n* = 231).

The patients with available gene expression data who were involved in our sub-study were representative of the entire population, and there were no substantial differences between their patient and tumour characteristics and patients not included (Table 1). The study participants provided written informed consent to allow further research analyses to be carried out on their tumour tissue. The primary end point of Fin-her, distant disease-free survival (DDFS), has been previously reported to be superior for the trastuzumab-containing arms after a median follow-up of 62 months [4].

The determination of hormone receptor status and HER2 expression by immunohistochemistry (IHC) was required locally and was performed according to the guidelines of each institution during the time of the study. Samples were considered hormone receptor positive if their level of oestrogen receptor (ER) and/or progesterone receptor (PR) was ≥ 10%. All patients with hormone positive tumours received five years of endocrine therapy. When HER2 expression was scored as 2+ or 3+ (on a scale of 0, 1+, 2+, or 3+), the number of copies of the HER2 gene was centrally determined by means of chromogenic *in situ* hybridisation (CISH) in reference laboratories.

The Responsify dataset (as part of a consortium supported by the European Commission under its Framework 7 Programme) is composed of 108 HER2-positive early stage breast cancer samples treated with adjuvant trastuzumab for one year, provided by Institut Jules Bordet (IJB) and Katholieke Universiteit Leuven (KUL). Gene expression, RPPA and clinical outcome data were available.

### Gene expression arrays

Of the available Fin-her HER2 samples, gene mRNA expression data was produced from 202 samples. All samples were re-evaluated to ensure tumour was present in the specimen. RNA was extracted from formalin-fixed, paraffin-embedded (FFPE) primary breast tumour tissue. Gene expression was measured using Affymetrix U219 GeneChips™ as per Affymetrix protocols on 96 well plates at AROS Applied Biotechnology A/S, Denmark. Affymetrix expression data were normalised using the RMA approach followed by a batch effect correction (affy (v.3.1.2) and SVA (v.3.10.0) packages of the R (v 3.1.2)/Bioconductor (v. 2.6) suite) [24]. When multiple probe sets mapped to the same official gene symbol, we computed the average value of their intensity. The Fin-her data is available at: http://www.ncbi.nlm.nih.gov/geo/query/acc.cgi?token=wfermmkijzktzcb&acc=GSE65095.

The Responsify expression dataset was produced using an Affymetrix HG-U133Plus2 platform at the J-C. Heuson Breast Cancer Translational Research Laboratory (BCTL) at IJB. Expression values were computed using the fRMA normalization method [25] (fRMA (v1.16.0)R/Bioconductor package). Again, when multiple probe sets mapped to the same official gene symbol, we computed the average value of their intensity. From the Responsify dataset, a total of 97 samples corresponding to 95 unique patients were processed. Appropriate quality assessments were conducted on the resulting files and 94 samples passed quality assurance for further analysis. The Responsify data is available at: http://www.ncbi.nlm.nih.gov/geo/query/acc.cgi?token=ulwbuuwgpzqphsh&acc= GSE58984.

Other gene expression datasets of expression profiles from HER2 positive breast tumors were retrieved from public databases or authors’ websites (previously described in [13]). To ensure comparability of expression values across multiple data sets we performed a 0.95 quantile normalization.

### Tumour infiltrating lymphocyte (TILs) evaluation

Evaluation of TILs was performed exactly as previously described [26, 27]. Two pathologists performed the readings independently and were blinded to clinical outcome; the mean value of two assessments was used for the current analyses. The correlation coefficient between the two pathologists was 0.77 (*p* < 0.001) and 0.49 (*p* < 0.001) for stromal and intratumoral TILs respectively.

### RPPA

Protein levels were assessed for the Responsify cohort in the laboratory of Professor Gordon Mills at the MD Anderson Cancer Center (Houston, TX) using RPPA as previously described [11]. Briefly, the tumour or cell lysates were diluted for and arrayed on nitrocellulose-coated slides. The samples were then probed with antibodies and visualised by colorimetric reaction. Finally, the slides were scanned and their density was quantified by Array-Pro.

### Computation of RPPA-based metagenes

We downloaded clinicopathological, normalised gene expression and RPPA data from the publicly available TCGA database using its online bioinformatics tools [11]. Eighty-seven samples with available gene expression and RPPA data were considered to be clinico-pathologically HER2-positive. Pathologically positive HER2 samples included those with HER2 IHC 3+ readings or a positive result on fluorescence *in situ* hybridization (FISH) for *HER2* amplification (HER2:Chr17 ratio ≥ 2). Each of the 141 RPPA assay samples available was assigned to one of two sample groups : the “low” or “high” groups of protein expression levels corresponding to the lower quartile or upper quartile of the expression level (Figure 2A). To identify the genes that were differentially expressed between low and high expression groups, we performed a differential gene expression analysis using a Student *t*-test with robust estimators of the central tendency and of the dispersion comparing high versus low RPPA expression tumours. 139 RPPA assays delivered differentially expressed metagenes (set of significantly differentially expressed genes [fdr ≤ 0.05]). Adjusted *p*-values (fdr) were computed using the Benjamini-Hochberg multi-testing correction method.

The expression levels of these metagenes in the gene expression datasets (Fin-her and Responsify) were computed using the metagene score described as follows:

Let *S* be a metagene (gene signature) composed of n genes (*s*1, …, *sn*) presenting a coefficient (-1 or 1, reflecting its down or up-regulation respectively). Let E be the set of expression values of the genes of *S* in one expression experiment. The metagene score (sigscore) is calculated by computing the sum of the products of the gene coefficient in the module (*s_i_*) by the corresponding gene expression value (*e_i_*) according to the following formula.

$$\text{Sigscore}=\sum_{i=1}^{n}e_{i}*s_{i}$$

The metagene score is the scalar product of the coefficient of the genes in the metagene and the gene expression values.

To assess the performances of the metagenes, we compute the metagenes score in the Responsify dataset and kept only the metagenes for which the score was highly correlated with the RPPA expression level (Pearson correlation index ≥ 0.5) and which achieved high predictive performances in ROC curves (fdr ≤ 0.05).

### Network representation of the intersecting metagenes

The network linking the intersecting metagenes (at least 5 common genes) was displayed with the yED software and data were clustered using the MCL graph clustering algorithm (http://www.yworks.com/en/products_yed_about.html) [28].

### Intersection between RPPA derived metagenes and functional classes

To determine whether the size of the intersection between a metagene sigA of size *Na* and a functional class sigB of size *Nb* was significant, we computed the hypergeometric *p*-value, which grossly corresponds to the probability of being wrong when estimating that having an intersection of size n genes is due to chance only when considering *N* genes in total.

$$\text{Pual}=P(X\geq n)=\sum_{i=n}^{\text{Nb}}\frac{\left(\begin{array}{c} \text{Na}\\ i \end{array}\right)\cdot \left(\begin{array}{c} N-\text{Na}\\ \text{Nb}-i \end{array}\right)}{\left(\begin{array}{c} N\\ \text{Nb} \end{array}\right)}$$

Where $\left(\begin{array}{c} x\\ y \end{array}\right)$ corresponds to the number of ways to choose *y* elements from a set of *x* elements. The *p*-value was then corrected using the Benjamini-Hochberg method.

These computations were performed using the compare-classes tool of the RSAT/Neat suite of tools [29]. This methodology was used to evaluate the ability of the model to identify groups of functionally related genes by comparing each metagene to the functional classes of the Gene Ontology and of mSigDB.

### Statistical analysis

For the outcome analysis, patients were scored according to each metagene and dichotomised at the median expression level. The primary outcome was distant disease free survival (DDFS), which was defined by the time interval between the date of randomisation and the date of first cancer recurrence outside of the ipsilateral/locoregional region or to death, whichever occurred first.

Patients alive at the last visit without documented evidence of distant metastases were censored. Associations between the different metagenes and ER status were investigated with a *t*-test, while the association among metagenes was estimated by computing the hypergeometric significance of their intersection. The Kaplan Meier method was used to generate survival curves, and the log-rank test was used to compare survival distributions. Univariate and multivariate models were computed using Cox proportional-hazards regression. Possible interactions with trastuzumab treatment were tested using a Wald test after adding a trastuzumab main effect and a product interaction term in the Cox model. Interaction effects were displayed using forest plots.

The Rand index is a metric ranging from 0 to 1 which describes the similarity between two clusters. We computed the Rand indices between the correlation matrices after applying a hierarchical clustering (Euclidian distance, complete linkage) and a partition into three classes with package flexclust (v 1.3–4) of R. Analyses were performed using the R statistical suite together with the genefu (v. 1.14.0), survival (v. 2.38–1) and rmeta (v. 2.16) Bioconductor packages. Our data were reported according to the essential elements of REMARK (reporting recommendations for tumour marker prognostic studies”) [30]. ROC curves and associated *p*-values were computed using the pROC (v1.8) package of R/ Bioconductor [31].

## SUPPLEMENTARY FIGURE, TABLES AND LEGENDS









## Abbreviations

ANXA1: AnnexinA1
DDFS: distant disease-free survival
FISH: fluorescence *in situ* hybridization
FFPE: formalin-fixed, paraffin-embedded
HER2: human epidermal growth factor receptor 2
HR: Hazard ratio
IHC: immunohistochemistry
Lck: lymphocyte-specific protein tyrosine kinase
Syk: Spleen tyrosine kinase
RPPA: reverse phase protein array
TCGA: tumour cancer genome atlas
