# Supplementary material for: Integrative proteomic and gene expression analysis identify potential biomarkers for adjuvant trastuzumab resistance: analysis from the Fin-her phase III randomized trial
Source: Oncotarget. 2015 Sep 3;6(30):30306–16. doi: 10.18632/oncotarget.5080 (PMC4745800; doi:10.18632/oncotarget.5080)
Supplement: Supplementary file 1 [file oncotarget-06-30306-s001.pdf]

SUPPLEMENTARY FIGURE, TABLES AND LEGENDS

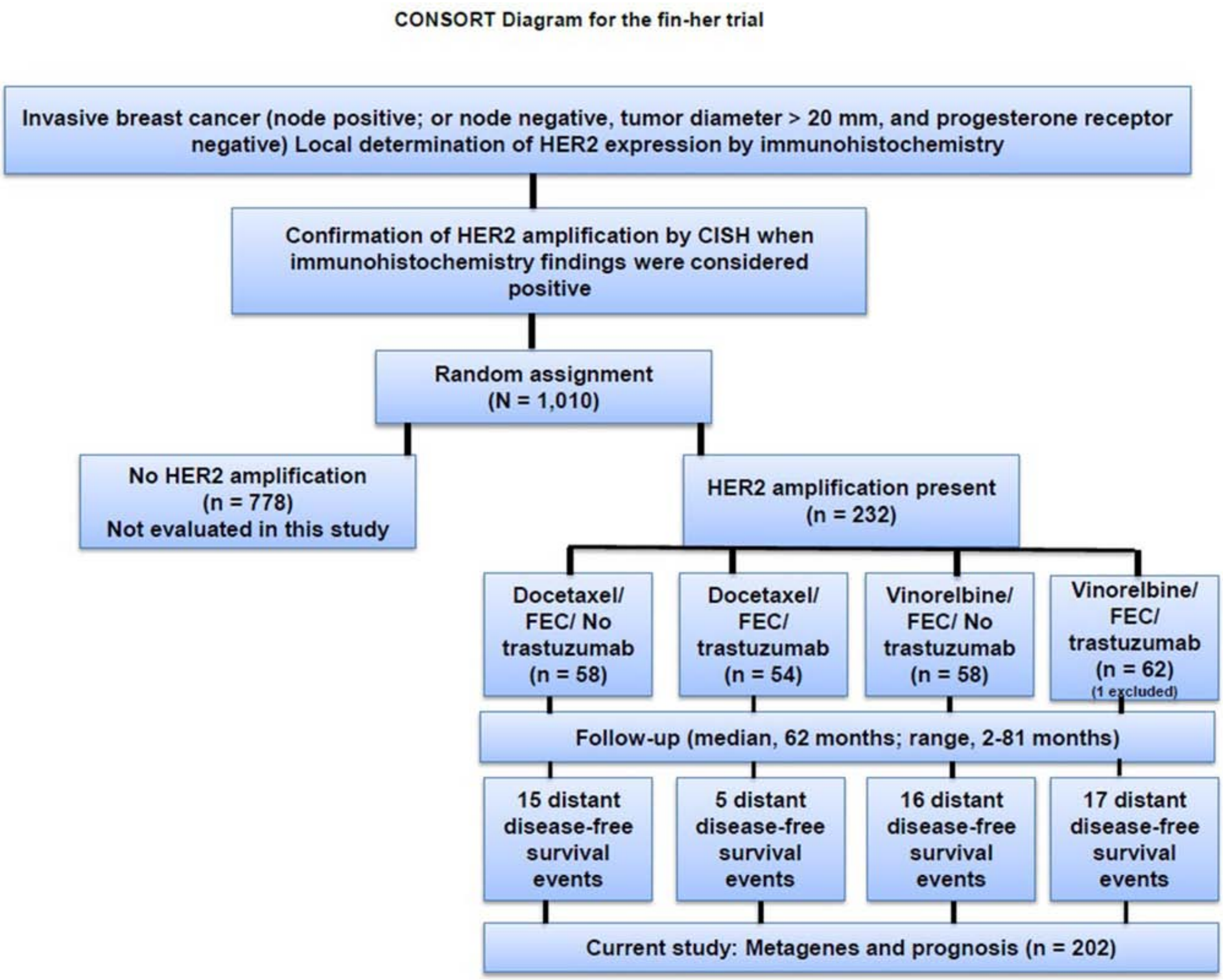

Supplementary Figure S1: CONSORT diagram of the fin-her trial.

Supplementary Table S1: Gene signatures that passed external validation.

	correlation R	AUC	AUC FDR
GATA3	0.802444568208424	0.918461538461538	1.5601428549056E-027
ER-alpha	0.676917979165032	0.806153846153846	0.0000243366625526168
Lck	0.640773427489008	0.758461538461538	0.00147874197520389
DJ-1	0.614607211758244	0.778461538461538	0.00041892051625558
Annexin_I	0.609487174567616	0.824615384615385	0.000000531216678363221
Caspase-7_cleavedD198	0.608719078173738	0.872307692307692	0.00000000103142117383112
Chk2	0.549234199491372	0.804615384615385	0.0000380175594917789
Notch1	0.545198878352904	0.773846153846154	0.000348502905380549
Bcl-2	0.529220569972019	0.829230769230769	0.000000410920636804509
Syk	0.5073541665497	0.776923076923077	0.000161221889728158

AUC- area under the curve
fdr- false discovery rate

Supplementary Table S2: List of the validated RPPA based signatures.

Supplementary Table S3: GO analysis for each of the 10 signatures.

Supplementary Table S4: MSigDB analysis for each of the 10 signatures.
